# Psychomotor Development and Traumatic Dental Injuries in Preschool

**DOI:** 10.1111/edt.13072

**Published:** 2025-06-19

**Authors:** Ednele Fabyene Primo‐Miranda, Maria Letícia Ramos‐Jorge, Rosane Luzia de Souza Morais, Paloma Christina Aguiar Silva, Débora Souto‐Souza, Leandro Silva Marques

**Affiliations:** ^1^ Departamento de Odontologia Universidade Federal dos Vales do Jequitinhonha e Mucuri (UFVJM) Diamantina Minas Gerais Brazil; ^2^ Departamento de Fisioterapia Universidade Federal dos Vales do Jequitinhonha e Mucuri (UFVJM) Diamantina Minas Gerais Brazil; ^3^ Departamento de Odontologia Centro Universitário Do Triângulo (UNITRI) Uberlândia Minas Gerais Brazil

**Keywords:** malocclusion, preschool children, psychomotor development, traumatic dental injury

## Abstract

**Background/Aim:**

Insufficient motor coordination can predispose children to falls and, consequently, the occurrence of traumatic dental injuries (TDIs). The present study investigated the association between psychomotor development and TDI in preschool children.

**Materials and Methods:**

A cross‐sectional study was conducted with 189 children aged three to six years in Diamantina, Brazil. Psychomotor development was assessed using the Test of Gross Motor Development—Second Edition (motor assessment) and a validated preschool version of the Mini‐Mental State Examination (cognitive assessment). Parents/guardians answered a socioeconomic questionnaire. Anthropometric measures were determined using WHO growth curves to obtain the body mass index (BMI). Intraoral examinations were performed by a single examiner trained and calibrated for diagnosing TDI based on the criteria proposed by Andreasen, with radiographic evaluations conducted when necessary. Lip coverage, anterior open bite, arch shape, and overjet were also recorded. Descriptive statistics, the chi‐square test, and Poisson regression (bivariate and multivariate) analyses were performed (95% CI, *p* < 0.05).

**Results:**

The prevalence of TDI was 50.3%. Delayed motor development (PR = 1.66; 95% CI: 1.25–2.22) and obesity (PR = 1.64; 95% CI: 1.10–2.45) were significantly associated with TDI in the adjusted model.

**Conclusion:**

Delayed motor development and obesity were associated with TDI in preschool children.

## 1 Introduction

Early childhood is a phase of learning and developing critical cognitive and motor skills [1, 2]. The motor performance of children contributes to the establishment of cognitive skills, such as memory, construction, and problem‐solving [3]. The prevalence of delayed motor development in children 12–60 months of age with typical cognitive development ranges from 3% to 11% [4]. Studies report that a low socioeconomic status, lack of space for play [3], obesity, screen time, and low physical activity may also be associated with delayed cognitive and motor development [5, 6, 7, 8, 9, 10, 11, 12].

Traumatic dental injury (TDI) poses a significant public health challenge, mainly due to its high prevalence, treatment costs, and long‐term effects on children's oral health [13, 14]. A systematic review with meta‐analysis found that the global prevalence of TDI in primary teeth is 24.2% [15]. The causes of TDI in these teeth are multifaceted, including local factors [15] (such as pronounced overjet, insufficient lip coverage, and anterior open bite), systemic factors (like epilepsy and cerebral palsy), and behavioral factors (including nonnutritive sucking habits and levels of physical activity) [15]. Given the complexity and variety of contributing factors [15], a multidisciplinary approach is essential to fully understand the causes of TDI in primary dentition.

Falls are one of the leading causes of TDI [16]. Difficulty in locomotion and controlling objects, imprecise movements, and slow reflexes make children more susceptible to falls, especially in school settings where they engage in frequent physical activity [2, 15, 16, 17]. Delayed motor development may lead to a greater occurrence of falls, increasing the risk of TDI [18, 19]. However, most studies on TDI fail to identify the cause of the fall, leading to subjective interpretations and unjustified extrapolations [2, 15, 16, 17]. Identifying the specific causes of falls in preschool children is crucial for establishing preventive measures.

Children with delayed psychomotor development may have greater difficulty with locomotion and protecting themselves during a fall, greater imprecision in their movements, and slowerreflexes than their classmates, making them more susceptible to falls during recreational activities at school [1, 18, 19]. Considering the interdependence between motor and cognitive development [4], one may hypothesize that delayed psychomotor development can lead to a deficient understanding of one's surrounding environment and greater proneness to falls and collisions, increasing the likelihood of TDI.

Knowledge of factors associated with falls in preschool children is essential to understanding potential risk relationships. The present study aimed to investigate the relationship between psychomotor development and the occurrence of TDI in preschool children.

## 2 Materials and Methods

This study was designed and written in accordance with the STROBE guidelines and received approval from the Human Research Ethics Committee.

A cross‐sectional study was conducted with a convenience sample of 189 children aged 3–6 years enrolled in two public daycare centers and a public school in Diamantina, located in the northeastern region of Minas Gerais, Brazil.

The final sample was composed of 189 children. The power of the sample was calculated for the main independent variables (TGMD2 and MMSE scores). The statistical power of the sample considering the TGMD2 score, 95% confidence level, 37 exposed children (delayed motor development), 152 nonexposed children (typical development), and frequency of TDIs of 78.4% and 43.4% for the two groups, respectively, was 96%. The statistical power of the sample considering the MMSE score, 95% confidence level, 106 exposed children (cognitive development below expected for age), 83 nonexposed children (cognitive development within the expected range for age), and frequency of TDIs of 58.5% and 39.8% for the two groups, respectively, was 72.6%.

The university's pediatric physiotherapy team performed the children's psychomotor assessment. The team comprised eight examiners trained in administering the Test of Gross Motor Development—Second Edition (TGMD‐2) [20] and the Mini‐Mental State Examination [21].

The TGMD‐2 is a test with individual norms for assessing motor development in children three to ten. The detailed assessment of movement requires an administration time of 20–30 min. The test comprises multiple fundamental motor skills, with the evaluation of the coordination of the torso and limbs during the performance of a motor skill.

The TGMD‐2 assesses twelve motor skills, divided into locomotion and object control. The locomotion skills include running, galloping, hopping, leaping, horizontal jumping, skipping, and sliding. The object control skills consist of two‐hand strike, bouncing, catching, kicking, overhand throwing, and rolling a ball. The test allows for separate evaluations of each subtest, with the ability to differentiate scores by gender for the object control skills. Total scores from each subtest are summed and presented as raw scores, which can then be converted into motor quotients [22].

The TGMD‐2 has been translated and validated for use on Brazilian children [20]. The sample was divided into two groups: delayed motor development (TGMD‐2 ≤ 88 points) and typical development (> 88 points).

Cognitive function was assessed using the Mini‐Mental State Examination (MMSE), which evaluates orientation, memory, attention, and verbal and written commands for 20–30 min [21]. For preschool‐aged children, the validated and adapted version of the MMSE was used, classifying them into two groups: those with a score below the expected range (≤ 23 points) and those within the expected range (> 23 points) [21].

BMI was calculated based on WHO growth curves, classifying children as obese (> 96th percentile), overweight (85th–96th percentile), or normal weight (3rd–84th percentile).

Socioeconomic status was determined using a questionnaire based on the classification of the Brazilian Association of Research Firms, which has a point system based on consumer goods and items in the home. The present study categorized family income as higher (Classes A and B) or lower (Classes C, D and E). The parents/guardians also answered a sociodemographic questionnaire addressing the sex and age of the child, the mother's schooling, and sucking habits, such as bottle feeding (absent or present), pacifier use (absent or present), and digit (finger or thumb) sucking (absent or present).

Dental variables were collected by a dentist (EFPM). Training for the diagnosis of malocclusion involved the use of images and plaster models. For the calibration exercise, 20 children were examined, and the results were compared to those of an experienced researcher who served as the gold standard (LSM) for determining interexaminer agreement using the Kappa statistic. After 10 days, the dentist conducted a second examination of the same children to assess the intraexaminer agreement. This follow‐up aimed to determine the consistency of the dentist's assessments over time. Kappa coefficients were ≥ 0.80 for all types of malocclusion. Training for the diagnosis of TDI involved the analysis of images and plaster models. For the calibration exercise, 20 children (nine with TDI and 11 without TDI) were examined, and the results were compared to those of an experienced researcher who served as the gold standard (MLRJ) to determine interexaminer agreement.

After 10 days, the same children were examined again to determine intraexaminer agreement, resulting in a Kappa coefficient of 0.90 for traumatic dental injuries (TDIs). A pilot study was conducted with 30 additional children and their parents or guardians.

The children were examined in a classroom under natural light. The teeth were first cleaned and dried with gauze. The clinical examination was exclusively visual and performed with a dental mirror, tongue depressor, endodontic file, and ruler to measure the overjet in millimeters (mm). Children whose trauma diagnosis was inconclusive based on clinical examination alone were referred for care at the UFVJM pediatric dentistry clinic, where they underwent radiographic examination.

TDI classification was based on Andreasen's criteria, and radiographic evaluations were conducted when necessary. Discoloration of the crown as a result of trauma was also recorded [23]. TDI was categorized as absent or present. The presence of malocclusion was defined according to the criteria proposed by Foster and Hamilton [24], and all the evaluations were performed with the teeth in occlusion. Overjet was measured as the distance from the palatal surface of the most prominent maxillary incisor to the corresponding mandibular incisor, classified as normal (< 3 mm) or accentuated (≥ 3 mm). The anterior open bite was defined as the absence of vertical overlap between the mandibular incisors. The dental arch shape was classified as normal or narrow. These evaluations were performed using a millimeter probe. Lip coverage was assessed in a relaxed environment without the child aware or performing any movement that could interfere with the assessment.

Data analysis was performed using the Statistical Package for the Social Sciences (SPSS version 25.0; SPSS Inc. Chicago, IL, USA). Descriptive statistics were performed for the variables of interest (TGMD‐2 score, MMSE score, BMI, sex, age, socioeconomic status, mother's schooling, digit sucking, use of a pacifier, bottle feeding, lip coverage, anterior open bite, accentuated overjet and arch shape). Comparisons between groups with and without TDI (dependent variable) were performed using the chi‐squared test and chi‐squared test for linear trend, with a *p* value ≤ 0.05 indicative of statistical significance. Bivariate and multivariate Poisson regression analyses were performed to calculate prevalence ratios (PR) and respective 95% confidence intervals (CI). The multivariate analysis was adjusted for all variables of interest. The significance level was set at 5% (*p* ≤ 0.05).

## 3 Results

The final sample was composed of 189 children. Table 1 displays the variables of interest (TGMD‐2 score, MMSE score, BMI, sex, age, socioeconomic status, mother's schooling, digit sucking, use of pacifier, bottle feeding, lip coverage, anterior open bite, accentuated overjet and arch shape) according to the absence/presence of TDI. The chi‐squared test, linear trend chi‐square test, and results of the unadjusted bivariate Poisson regression analysis revealed that the TGMD‐2 score (*p* ≤ 0.01), MMSE score (*p* = 0.01), BMI (*p* = 0.02), inadequate lip coverage (*p* = 0.03), anterior open bite (*p* = 0.00), accentuated overjet (*p* = 0.00), and arch shape (*p* = 0.00) were associated with the presence of TDI.

**TABLE 1:** Distribution of characteristics of preschool children according to absence/presence of traumatic dental injury and results of unadjusted Poisson regression analysis (*n* = 189, Diamantina, Brazil).

Variables	Traumatic dental injury				
	Absent *n* (%)	Present *n* (%)	^a^ *p*	Unadjusted PR (95% CI)	*p*
*TGMD‐2*					
Typical development Motor delay	86 (56.6) 8 (21.6)	66 (43.4) 29 (78.4)	**< 0.001**	1 1.80 (1.41–2.31)	**< 0.001**
Mini‐Mental State					
Within the expected age	50 (60.2)	33 (39.8)		1	
Below expected age	44 (41.5)	62 (58.5)	**0.010**	1.47 (1.08–2.00)	**0.010**
BMI					
Normal	74 (55.6)	59 (44.4)		1	
Overweight	16 (37.2)	27 (62.8)		1.41 (1.05–1.91)	**0.022**
Obesity	4 (30.8)	9 (69.2)	**0.011** ^b^	1.56 (1.04–2.35)	**0.031**
Child's sex					
Male	48 (52.7)	43 (47.3)		1.00	
Female	46 (46.9)	52 (53.1)	0.422	1.12 (0.84–1.49)	0.430
Child's age					
5–6 years	66 (48.9)	69 (51.1)		1.00	
3–4 years	28 (51.9)	26 (48.1)	0.710	0.94 (0.68–1.30)	0.721
Family income					
Classes A, B Classes C, D, E	33 (51.6)	31 (48.4)		1.00	
	61 (48.8)	64 (51.2)	0.720	1.06 (0.78–1.43)	0.720
Mother's schooling					
≥ 11 years of study	66 (48.9)	69 (51.1)		1.00	
< 11 years of study	28 (51.9)	26 (48.1)	0.711	0.94 (0.68–1.30)	0.721
Digit sucking					
*No*	89 (50.6)	87 (49.4)		1	
*Yes*	5 (38.5)	8 (61.5)	0.402	1.24 (0.79–1.96)	0.340
Use of pacifier					
No	82 (47.4)	91 (52.6)		1	
Yes	12 (75.0)	4 (25.0)	**0.030**	0.47 (0.20–1.12)	**0.092**
Bottle feeding					
No	87 (49.4)	89 (50.6)		1	
Yes	7 (53.8)	6 (46.2)	0.764	0.91 (0.49–1.67)	0.773
Lip coverage					
Adequate	92 (52.0)	85 (48)		1	
Inadequate	2 (16.7)	10 (83.3)	**0.021**	1.73 (1.29–2.33)	**< 0.001**
Anterior open bite					
Absent	92 (52.6)	83 (47.4)		1	
Present	2 (14.3)	12 (85.7)	**0.010**	1.81 (1.39–2.35)	**< 0.001**
Overjet					
< 3 mm	89 (53.6)	77 (46.4)		1	
≥ 3 mm (accentuated)	5 (21.7)	18 (78.3)	**0.001**	1.69 (1.29–2.21)	**< 0.001**
Arch shape					
Normal	93 (51.70	87 (48.3)		1	
Narrow	1 (11.1)	8 (88.9)	**0.021**	1.84 (1.39–2.42)	**< 0.001**

*Note:* Significance of bold values indicate statistical significant of *P* value *p* < 0.05.

^a^ Chi‐squared test.

^b^ Chi‐squared test for linear trend.

After adjusting the variables in the multivariate Poisson regression analysis (Table 2), motor development remained significantly associated with TDI, whereas cognitive function lost statistical significance. Children with delayed motor development were 66% more likely to have TDI (PR = 1.66; 95% CI: 1.25 and 2.22) than those with typical development. Children with obesity were 64% more likely to have TDI (PR = 1.64; 95% CI: 1.10–2.45) compared to those with a normal BMI. The socioeconomic variables, harmful oral habits, and occlusal characteristics were not associated with TDI (Table 2).

**TABLE 2:** Results of adjusted Poisson regression analysis of factors associated with traumatic dental injuries in preschool children (*n* = 189, Diamantina, Brazil).

Variables	Adjusted PR (95% CI)	*p
TGMD‐2		
Typical development	1	**0.001**
Motor delay	1.66 (1.25–2.22)	
Mini‐Mental State		
Within the expected age	1	
Below the expected age	26 (0.91–1.74)	0.174
BMI		
Normal	1	
Overweight	1.32 (0.97–1.79)	0.082
Obesity	1.64 (1.10–2.45)	**0.01**
Use of pacifier		
No	1	
Yes	0.51 (0.22–1.17)	0.111
Lip coverage		
Adequate	1	
Inadequate	0.86 (0.52‐1.42)	0.562
Anterior open bite		
Absent	1	
Present	1.16 (0.67–1.99)	0.593
Overjet		
< 3 mm	1	
≥ 3 mm (accentuated)	1.45 (0.73–2.86)	0.282
Arch shape		
Normal	1	0,604
Narrow	1.18 (0.64–1.99)	

*Note:* *Multivariate Poisson regression analysis adjusted for TGMD‐2 score, Mini‐Mental State Examination score, BMI, sex, age, socioeconomic status, mother's schooling, digit sucking, pacifier use, bottle feeding, lip coverage, anterior open bite, accentuated overjet and arch form. Significance of bold values indicates statistical significant of *P* value *p* < 0.05.

Abbreviations: CI, confidence interval; PR, prevalence ratio calculated using Wald's chi‐squared test.

## 4 Discussion

The results of the present study showed that delayed motor development predisposes children to traumatic dental injuries (TDIs). Children with delayed motor development were 66% more likely to have TDI than children with typical development. This is the first study to consider delayed motor development as an etiological factor for the occurrence of TDIs in preschool children.

Studies indicate that adequate motor performance for a child's age plays a crucial role in developing cognitive skills, with associations found between cognitive development and motor skills in childhood [25, 26, 27]. Given the interdependence of mental and motor development, the main objective of this study was to investigate the relationship between psychomotor development and TDI. Cognitive development was associated with TDI in the unadjusted analysis, but this significance was lost in the adjusted analysis. The interdependence between the two variables may have influenced this result, revealing a more significant influence of motor development on the occurrence of TDI in children 3–6 years of age. Motor development is considered a predictor of cognitive development [25, 26, 27], which may explain the result of the present study.

Among the primary tests used to assess gross motor development, the TGMD‐2 is unique in that it evaluates gross motor skills separately, emphasizing the quality of performance for each skill [20]. Previous studies have shown that test–retest reliability is higher for the TGMD‐2 than the main tests used to assess gross motor skills in children with typical development [25]. In the present investigation, psychomotor development (TGMD‐2 and MMSE) was assessed by an experienced physiotherapy team (made up of physiotherapists and a psychologist) that had undergone training and calibration exercises, enhancing the study's internal validity. Studies have shown that falls due to the imprecision of movements in children constitute one of the main etiologies of TDI in deciduous teeth [15, 16, 17]. However, the cause of falls is not normally investigated.

The school setting is a space for exploring games and is where children spend much time. Delayed motor development could predispose children to falls and increase the likelihood of TDI. Thus, a more in‐depth investigation of the causes of falls in preschool children should be performed so that the causality between motor development and TDI can be clarified better.

The high prevalence of TDI in the sample (50.3%) may be because this is not an epidemiological study with a representative sample and because the diagnosis was made using radiography in cases of doubt during the clinical examination. Clinical factors, such as excess weight and obesity, remained associated with TDI in the adjusted analysis. Studies have shown that the prevalence of TDI is higher in children with obesity compared to those in the ideal weight range [12]. Excess weight can cause imbalance and postural instability in children [28]. Thus, the association between obesity and proneness to falls seems to be causal, as weight loss has been proven to increase postural stability [27, 29], which may explain the association found in the present study.

Accentuated overjet, a narrow dental arch, anterior open bite, and inadequate lip coverage were associated with TDI in the unadjusted analysis but lost statistical significance after adjusting all variables in the multivariate analysis. Thus, behavioral factors exert a stronger influence than clinical factors on the predisposition to TDI in the present sample of children.

The cross‐sectional design of this observational study limits the ability to infer causality, so the results should be interpreted cautiously. Longitudinal studies are necessary to establish the cause‐and‐effect relationship between psychomotor development and TDI in preschool children. These longitudinal studies may benefit from a hierarchical conceptual framework to guide the multivariate analysis. This approach allows for separating variables at different levels (e.g., individual versus contextual factors) and could provide a clearer understanding of the pathways influencing TDI risk.

Identifying delayed psychomotor development and understanding its relationship with TDI can contribute to a change in public health strategies and the prevention of TDI. This relationship draws attention to assessing psychomotor development in preschool children with greater frequency and attention. The prevention and early intervention of these conditions can contribute to the adequate development of children for age and avoid health problems.

## 5 Conclusion

Delayed motor development and obesity were significantly associated with a higher occurrence of TDI in preschool children. Dental clinicians could play a proactive role by observing early signs of motor delays and collaborating with pediatricians for at‐risk children.

## Author Contributions

Ednele Fabyene Primo‐Miranda: data collection, bibliographic research, statistical analysis and article writing. Rosane Luzia de Souza Morais, Débora Souto‐Souza, Paloma Christina Aguiar Silva: writing the article and bibliographic research. Leandro Silva Marques and Maria Letícia Ramos‐Jorge: study design; analysis and interpretation of data for work; critical review of important intellectual content.

## Ethics Statement

This study was designed and written in accordance with the STROBE guidelines and received approval from the Human Research Ethics Committee (protocol number: 1.604.987) of the Federal University of the Vales do Jequitinhonha e Mucuri (UFVJM) in Diamantina, Minas Gerais, Brazil.

## Conflicts of Interest

The authors declare no conflicts of interest.

## Data Availability

The data that support the findings of this study are available from the corresponding author upon reasonable request.
